# *Withania somnifera* Reverses Transactive Response DNA Binding Protein 43 Proteinopathy in a Mouse Model of Amyotrophic Lateral Sclerosis/Frontotemporal Lobar Degeneration

**DOI:** 10.1007/s13311-016-0499-2

**Published:** 2016-12-07

**Authors:** Kallol Dutta, Priyanka Patel, Reza Rahimian, Daniel Phaneuf, Jean-Pierre Julien

**Affiliations:** 1 Centre de Recherche de l’Institut Universitaire en Santé Mentale de Québec, Québec City, G1J 2G3 Canada; 2 Present Address: Department of Biological Sciences, University of South Carolina, Columbia, SC 29208 USA; 3 Department of Psychiatry and Neuroscience, Université Laval, Québec City, G1V 0A6 Canada

**Keywords:** Amyotrophic lateral sclerosis, *Withania somnifera*, TDP-43, NF-κB.

## Abstract

Abnormal cytoplasmic mislocalization of transactive response DNA binding protein 43 (TARDBP or TDP-43) in degenerating neurons is a hallmark of amyotrophic lateral sclerosis (ALS) and frontotemporal lobar degeneration with ubiquitin-positive inclusions (FTLD-U). Our previous work suggested that nuclear factor kappa B (NF-κB) may constitute a therapeutic target for TDP-43-mediated disease. Here, we investigated the effects of root extract of *Withania somnifera* (Ashwagandha), an herbal medicine with anti-inflammatory properties, in transgenic mice expressing a genomic fragment encoding human TDP-43^A315T^ mutant. Ashwagandha extract was administered orally to hTDP-43^A315T^ mice for a period of 8 weeks starting at 64 and 48 weeks of age for males and females, respectively. The treatment of hTDP-43^A315T^ mice ameliorated their motor performance on rotarod test and cognitive function assessed by the passive avoidance test. Microscopy examination of tissue samples revealed that Ashwagandha treatment of hTDP-43^A315T^ mice improved innervation at neuromuscular junctions, attenuated neuroinflammation, and reduced NF-κB activation. Remarkably, Ashwagandha treatment reversed the cytoplasmic mislocalization of hTDP-43 in spinal motor neurons and in brain cortical neurons of hTDP-43^A315T^ mice and it reduced hTDP-43 aggregation. *In vitro* evidence is presented that the neuronal rescue of TDP-43 mislocalization may be due to the indirect effect of factors released from microglial cells exposed to Ashwagandha. These results suggest that Ashwagandha and its constituents might represent promising therapeutics for TDP-43 proteinopathies.

**Electronic supplementary material:**

The online version of this article (doi:10.1007/s13311-016-0499-2) contains supplementary material, which is available to authorized users.

## Introduction

Transactive response DNA binding protein was originally described as a regulatory element involved in modulating HIV-1 gene expression [1]. The 414 amino acid-containing protein has a molecular weight of 43 kDa (hence it is commonly referred to as TDP-43), and structurally consists of 2 RNA recognition motifs, a nuclear localization sequence, a nuclear export domain, and a glycine-rich C-terminal domain [2]. TDP-43 is predominantly a nuclear protein, even though it is capable of shuttling between the nucleus and cytoplasm—a process partly regulated by nuclear localization signal and nuclear export signal motifs [3]. TDP-43 is a DNA/RNA binding protein and it is involved in regulating RNA transcription, splicing, trafficking, and microRNA biogenesis [4]. Its involvement in neurodegenerative disorders was first reported in 2006 when hyperphosphorylated, ubiquitinated, and cleaved C-terminal fragments of the protein were detected from postmortem brain and spinal cord of patients suffering from frontotemporal lobar degeneration with ubiquitin-positive inclusions (FTLD-U) and amyotrophic lateral sclerosis (ALS) [5, 6]. TDP-43 has been shown to be prone to aggregation [7], and neuronal and glial TDP-43 inclusions have since been reported from >95% of sporadic ALS cases. Protein cleavage, aggregation, and neurotoxicity enhancing mutations in the *TARDBP* have also been reported from both familial and sporadic ALS cases [8].

Cellular levels of TDP-43 appear to be tightly regulated. The protein has the intrinsic property of autoregulating its RNA level by binding with the 3' untranslated region leading to the excision of an intron, thereby resulting in its degradation by nonsense-mediated RNA decay [9, 10]. However, in some human patients it has been demonstrated that TDP-43 levels are elevated [11, 12], suggesting that disease-associated TDP-43 aggregates disrupt its self-regulation, thereby contributing to the pathogenesis. Mislocalization of nuclear TDP-43 into the cytoplasm is also an early event in disease pathology, resulting in neurotoxicity [13]. However, whether nuclear depletion, constituting a “loss of function” of the protein, or its accumulation in cytoplasm constituting a “novel gain of toxic function” plays the key role in the disease has been a matter of debate [13, 14]. Nonetheless, insights from few recent studies favor the “loss of nuclear function” hypothesis [15–19].

In a previous study, we provided evidence that nuclear factor kappa B (NF-κB) may constitute a therapeutic target in ALS pathogenesis with TDP-43 deregulation. We showed that TDP-43 binds to and acts as a coactivator of the P65 subunit of NF-κB [12]. Postmortem spinal cord samples from sporadic ALS cases exhibited elevated levels of NF-κB mRNA when compared with age-matched controls and the p65 NF-κB protein displayed abnormal nuclear localization in neurons. In addition, treatment of transgenic mice expressing human TDP-43^A315T^ mutant with Withaferin A, a NF-κB inhibitor, ameliorated disease phenotypes. Withaferin A is a component of the plant *Withania somnifera dunal* [or Ashwagandha (ASH)], a perennial plant belonging to the family Solanaceae [20] that has been in use for the last 4000 years in traditional Indian medical system (*Ayurveda*). The root of the plant reportedly contains 14 to 15 different alkaloids and about 40 structurally similar steroidal lactones (called withanolides) other than various carbohydrates and amino acids, in varying amounts [21]. It reportedly has antimicrobial, anti-inflammatory, antineoplastic, antistress, cardioprotective, antidiabetic, and neuroprotective properties [22]. The neuroprotective property of the plant extract or purified products from the plant has been demonstrated in multiple disease models such as Alzheimer’s disease, Parkinson’s disease, Huntington’s disease, and spinal cord injuries [23]. The current study was designed to evaluate the efficacy of Ashwagandha (ASH) root extract to ameliorate behavioral and pathological phenotypes in a transgenic mouse model of ALS/FTLD exhibiting TDP-43 proteinopathy [24].

## Materials and Methods

### Preparation of ASH Root Extract

*Withania somnifera* (ASH) root, rich in various withanolides and alkaloids, was provided to us in dried powder form by Valeant Pharmaceuticals International Inc (Quebec, Canada). The product is an 11:1 extract from the plant root.

### ALS/FTLD Mouse Model and Treatment Paradigm

A transgenic mouse line bearing human genomic fragment encoding TDP-43 with A315T mutation was generated previously by us [24]. Animals were randomly distributed in either ASH-treated (*n* = 28) or Vehicle (Veh)-treated (*n* = 24) groups. The average age of the male mice at the beginning of treatment was 445 ± 0.45 days and that of female mice was 338 ± 3.2 days. Birth records of all the mice used for the experiments are provided in Figure S1. Mice in the ASH group were fed 5 mg root powder (by gavage) as a suspension in 200 μl sterile buffered saline every alternate day, for 8 or 16 weeks. Animals in the Veh group received equal volumes of buffered saline only for the same durations. This treatment had no effect on survival and body weight of the animals, and there were no outward manifestation of deleterious side effects. The Animal Care Ethics Committee of Université Laval approved all *in vivo* experimental protocols. Experiments were carried out in accordance with the Guide to the Care and Use of Experimental Animals of the Canadian Council on Animal Care.

### Rotarod Performance Test

To test motor coordination in treated and untreated mice they were allowed to run on an accelerating rotarod at 3 rpm speed with 0.25 rpm/s acceleration. Mice were subjected to 3 trials per session every week and the longest latency to fall from the rotating rod was recorded. The maximum cut-off limit was set for 3 min.

### Passive Avoidance Test

One-trial passive avoidance test was performed as described earlier [25], with minor modifications. The latency time for mice to enter the dark compartment was measured, with a 5-min cut-off.

### Immunofluorescence Microscopy and Image Analysis

Spinal cord and brain samples collected postsacrifice were sectioned and subjected to immunofluorescent staining by methods previously described [26]. Details of antibodies used are provided in Table 1. Slides were visualized under a Zeiss Apotome or Leica DM5000B microscope. To quantify nuclear or cytoplasmic staining intensity, unprocessed single-channel images were overlaid with 4,6-diamidino-2-phenylindole (DAPI) with the help of Adobe Photoshop CS5. The nuclear area was traced based on the DAPI signal with the pencil tool followed by tracing of the entire cell body, also with the pencil tool, but with a different color. All the cells of interest were thus marked followed by removal of the DAPI channel and the images were saved. Further analysis of images were carried out by following a protocol described elsewhere [27], with minor modifications. Briefly, the images were opened with ImageJ software and converted to 16-bit. The regions of interest (already marked out in Photoshop) were selected using the freeform selection tool. Measurement was set for integrated density from the Analyze menu. Whole cell and/or its nuclear fluorescence intensities were measured after correcting for background fluorescence. Nuclear:cytoplasmic ratio or just nuclear fluorescence intensity or overall fluorescence intensity was calculated from data generated from multiple sections.

**Table 1:** List of antibodies used for Western blots (WB) and immunofluorescence (IF)

Antibody against	Dilution for WB	Dilution for IF	Company
Actin	1:10,000		Millipore (Temecula CA, USA)
Arginase 1	1:1000		Santa Cruz (Santa Cruz, CA, USA)
Cox-2	1:1000		Cell Signaling Technologies (Danvers, MA, USA)
Glyceraldehyde 3-phosphate dehydrogenase	1:2000		Santa Cruz
Glial fibrillary acidic protein	1:5000	1:300	Cell Signaling Technologies
Hemagglutinin antigen HA	1:1000		Roche Applied Sciences (Penzberg, Germany)
Human transactive response DNA binding protein 43 (TDP-43; clone 2E2-D3)	1:1000	1:200	Abnova (Taipei City, Taiwan)
Iba-1		1:500	Wako Chemicals (Richmond, VA, USA)
IkBa	1:1000		Santa Cruz
Inducible nitric oxide synthase	1:1000		BD Biosciences (San Jose, CA, USA)
NeuN		1:500	Cell Signaling Technologies
Neurofilament-H		1:100	Millipore
Nuclear factor kappa B (NF-κB)	1:1000		Santa Cruz
P84	1:2500		Abcam (Cambridge, UK)
Pan-TDP-43	1:5000		Proteintech (Chicago, IL, USA)
Peripherin	1:5000		Millipore
Phospho-NF-κB (Ser 536)		1:250	Cell Signaling Technologies,
Synaptic vesicle protein-2 SV-2		1:25	Developmental Studies Hybridoma Bank (Mt. Prospect, IA, USA)
Tumor necrosis factor-α	1:400		Abcam
Ym-1	1:500		Stem Cell Technologies (Vancouver, Canada)

### Neuromuscular Junction Staining

Twenty-μm-thick cryosections of mouse gastrocnemius muscle were used to stain for neuromuscular junctions (NMJs). Presynaptic connections were stained using neurofilament-H and synaptic vesicle protein 2 (SV2). Tetramethylrhodamine-conjugated α-bungarotoxin (1:100; Sigma-Aldrich, St. Louis, MO, USA) was used to label acetylcholine receptors located at the subsynaptic membrane. Montages of z-stack images were captured using a Zeiss Apotome microscope and the images were processed using ImageJ and Adobe Photoshop. NMJs were classified as fully innervated, partially innervated, or denervated, based on the extent of overlap of α-bungarotoxin and neurofilament-H+SV2 staining.

### Protein Extraction Protocols

Spinal cords were excised from anesthetized mice after perfusion with ice-cold 0.9% saline. Whole protein lysates were extracted by methods previously described [26]. Cytosolic and nuclear fractions from cultured cells were also prepared as per methods described elsewhere [28]. Purity of the fractions was confirmed by the presence of cytosolic glyceraldehyde 3-phosphate dehydrogenase or nuclear P84 proteins.

### Immunoblot Analysis

Immunoblot analysis were performed from samples containing equal amount of protein (quantified by Bio-Rad Protein assay; Bio-Rad, Hercules, CA, USA) by methods previously described [26]. Details of antibodies used are represented in Table 1.

### Generation of Stable NSC34-hTDP^A315T^ Cell Line

For *in vitro* experiments, NSC34 (a murine neuroblastoma/spinal cord hybrid) cell line was used. Mammalian expression vector plasmid pCMV–TDP-43 with point mutation A315T and a HA tag was generated previously [12]. Cells were transfected with the pCMV–TDP-43^A315T^ plasmid using Lipofectamine 2000 reagent (Life Technologies, Carlsbad, CA, USA) and were subsequently selected in Dulbecco’s modified eagle medium (DMEM) containing 375 μg/ml Geneticin (G418; Life Technologies). For further experiments, selected clones were amplified, checked for protein expression, and propagated. Prior to experimentation, these cells were differentiated as per the published protocol [29].

### Luciferase Assay to Check p65 Activation in Microglia

Mouse microglial cell line BV2 was stably transfected with pGL4.32[*luc2P/*NF-κB–RE/Hygro] plasmid DNA (Promega, Madison, WI, USA). The pGL4.32[*luc2P/*NF-κB–RE/Hygro] vector contains 5 copies of an NF-κB response element that drives transcription of the luciferase reporter gene *luc2P*. The stable cell line was maintained in DMEM supplemented with 10% fetal bovine serium and 100 μg/ml hygromycin. Cells (5 × 10^4^ per well) were seeded in 24-well plates. The cells were stimulated with 100 ng/ml bacterial lipopolysaccharide (LPS) for 3 h, after which the media was removed, wells washed with 1× PBS, followed by cell lysis using Glo Lysis buffer (Promega). Luciferase activity was measured using the Bright-Glo Luciferase assay system (Promega), according to the manufacturer’s instructions. To test the efficacy of ASH on reducing LPS-induced P65 activity, cells were treated with varying concentrations of ASH extract in dimethyl sulfoxide (DMSO; 1 μg, 10 μg, 100 μg, 250 μg, and 500 μg per ml) for 3 h post-LPS treatment, followed by luciferase assay. Control studies were done with cells treated with ASH alone. Results were expressed as mean of luciferase activity/μg cellular protein from at least 6 wells in each treatment condition.

### Cell Survival Assay

Cell viability post-ASH treatment was assessed using [3-(4,5-dimethylthiazol-2-yl)-5-(3-carboxymethoxyphenyl)-2-(4-sulfophenyl)-2H-tetrazolium] MTS assay, as per the manufacturer’s instructions (Promega). BV2 was seeded onto 96-well plates at a density of 10^4^ cells/well. The treatment paradigm was similar to that explained for luciferase assay. Postincubation with MTS reagent, the absorbance, reflecting the reduction of MTS by viable cells, was determined at 490 nm using an EnSpire 2300 Multilabel reader (Perkin Elmer, Waltham, MA, USA). Values were expressed as a percentage relative to those obtained in controls.

To test the effect of ASH extract on viability of NSC34–hTDP-43^A315T^, cells were seeded in a 96-well plate and subsequently treated with 250, 100, 10, and 1 μg/ml ASH extract for 6 h. Values were expressed as a percentage relative to those obtained in controls.

### *In Vitro* Model of Inducing hTDP-43 Mislocalization

To induce mislocalization of nuclear TDP-43 into the cytoplasm in NSC34–hTDP-43^A315T^ cells, cells were subjected to excitotoxic (2 mM glutamate in buffer containing 125 mM NaCl, 10 mM CaCl_2_ 5.9 mM KCl, 11.6 mM HEPES, and 11.5 mM glucose; pH 7.4), inflammatory [40 ng/ml of recombinant tumor necrosis factor (TNF)-α], or oxidative stimuli (50 μM) for varying time points [29]. Cells were collected, washed once in ice-cold 1× PBS, and subjected to fractionation for separation of cytosolic and nuclear fractions as described above. Immunoblotting was performed with the protein samples to determine the level TDP-43 in each fraction. Untreated NSC34–hTDP-43^A315T^ served as control.

### Effect of Microglia-Conditioned Media on Glutamate-Stimulated NSC34–hTDP-43^A315T^

To test the role of soluble factors released from microglia on TDP-43 distribution in NSC34–hTDP-43^A315T^ cells, BV2 cells were seeded at a density of 2 × 10^6^ cells per 10-cm plate and cultured from 16–20 h in DMEM containing 10% FBS. Prior to treatment, the culture media was replaced with serum-free DMEM. Subsequently, the cells were treated with either LPS (100 ng/ml) or ASH (250 μg/ml dissolved in DMSO) and incubated for 4 h at 37 °C. Control BV2 cells were treated with an equal volume of DMSO only. After 4 h the culture supernatant was collected and centrifuged under sterile conditions at 2655 g for 5 min to precipitate cellular debris; this served as the conditioned media. NSC34–hTDP-43^A315T^ cells that had been seeded separately into 3 other plates had been treated with glutamate (as described above) almost at the same time as BV2 treatment. Prior to addition of the conditioned media onto the NSC34–hTDP-43^A315T^ cells, their glutamate-containing media were removed, plates were washed with sterile PBS, and finally incubated with the BV2-conditioned media and some fresh serum-free DMEM (at a ratio of 2:1) for 12 h. Postincubation, cells were collected and subjected to nuclear cytosolic fractionation. Subsequently, they were analyzed by immunoblotting to determine TDP-43 levels. As negative controls for this experiment, glutamate-treated NSC34–hTDP-43^A315T^ cells were treated with BV2-conditioned media that had been heated at 70 °C to denature any soluble factors that may have been contained in them.

### Culture and Treatment of Primary Mouse Microglia

Primary microglia were cultured from the brains of pups (postnatal day 6) of TDP-43^A315T^ mutant mice. Brains were collected and placed in ice-cold PBS. Following mechanical dissociation, the brains were incubated in a 0.25% Trypsine-EDTA solution (Sigma-Aldrich) containing 250 K U/ml DNase I (Sigma-Aldrich). After centrifugation, the cell pellets were placed in T-75 cm^2^ flasks (Sarstedt, Nümbrecht, Germany) for 10 d at 37 °C, 5% CO_2_, in DMEM high-glucose media with 10% fetal bovine serum and antibiotic solution (Sigma-Aldrich). Genotyping carried out from tissues of the pups confirmed the identity of each culture. At confluence, the wild-type (WT), as well as transgenic microglia, were plated in 6-well plates at a concentration of 200,000 cells/well in serum containing media. Cells were incubated with granulocyte colony-stimulating factor 24 h later to allow adhesion. After 2 to 3 days cells were deemed suitable for experimentation.

Prior to treatment, cells were transferred to serum-free media. Microglia from WT mice or transgenic TDP-43^A315T^ mice were challenged with LPS or DMSO for 6 h to elicit cytokine/chemokine release. One set of WT microglia was treated with LPS for 3 h followed by further addition and incubation with ASH extract in DMSO (250 μg/ml) for 3 h more. Postincubation, the media was collected, centrifuged at 1000 × g for 5 min to precipitate out any debris, and the resultant media was used for estimation of cytokine/chemokine release.

### Cytokine Array

The cytokine expression profiles from primary microglia at different treatment conditions were performed with mouse cytokine antibody array kit (Raybio Mouse Inflammation Antibody Array 1, Cat#AAM- INF-1; RayBiotech, Norcross, GA, USA) as previously described [26]. Cell culture media post-treatment were centrifuged at 300 × g to remove cellular debris and incubated with the array membranes overnight at 4 °C. After washing in with the buffer provided with the kit, membranes were incubated with biotin-conjugated antibodies overnight. Signal detection was performed according to the RayBiotech protocol, by exposing membranes to X-ray film (Biomax MR1; #8701302; Kodak, Rochester, NY, USA), and the obtained results analyzed using ImageJ software. Data are expressed in arbitrary units relative to appropriate positive controls.

### Effect of Direct ASH Treatment on NSC34–hTDP-43^A315T^ Cells

To evaluate if ASH can directly affect TDP-43 re-distribution, NSC34–hTDP-43^A315T^ cells were treated with ASH at a concentration of 10 μg/ml, with or without glutamate pretreatment. TDP-43 levels were determined by immunoblotting from nuclear and cytoplasmic fractions of these cells.

### Statistical Analysis

Prism 5.0 (GraphPad, La Jolla, CA, USA) was used for all statistical analysis. Comparisons between 2 groups were done by unpaired two-tailed *t* test with Welch’s correction. Comparison between multiple groups was done by 1-way analysis of variance with Bonferroni’s post-test. A *p*-value up to 0.05 was considered significant.

## Results

### ASH Extract Inhibits NF-κB Activation in Cultured Cells

**Fig. 1:**
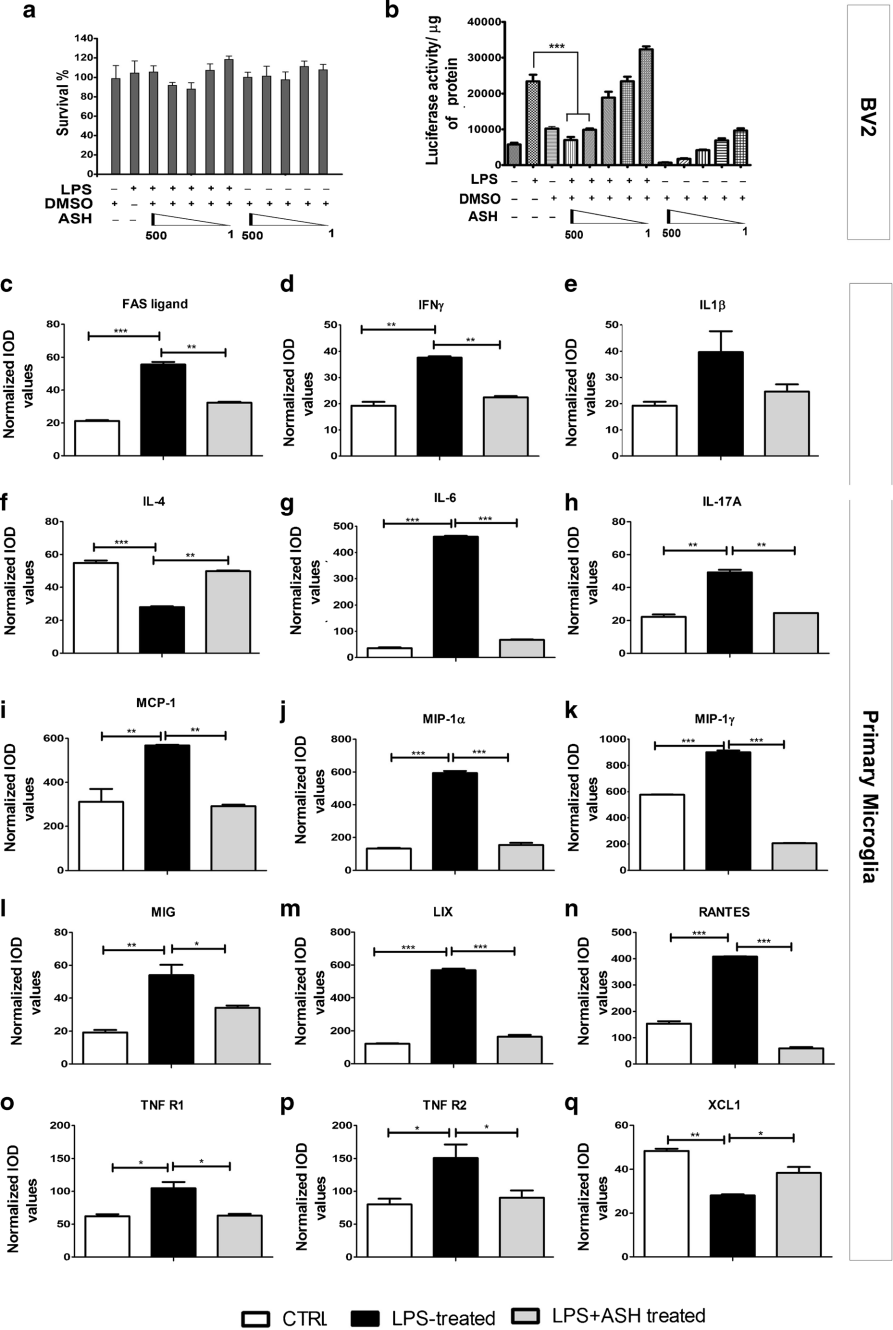
Effective inhibition of nuclear factor kappa B (NF-κB) activity by Ashwagandha (ASH) extract in mouse microglial BV2 cells and modulation of cytokine/chemokine activity in primary microglia. No significant alteration in BV2 cell survival was observed by addition of lipopolysaccharide (LPS) or ASH extract in dimethyl sulfoxide (DMSO) at varying concentrations (500, 250, 100, 10, and 1 μg/ml) (**a**). BV2 cells were stably transfected with pGL4.32[*luc2P/*NF-κB–RE/Hygro] plasmid carrying 5 copies of an NF-κB response element that drives transcription of the luciferase reporter gene *luc2P.* When treated with bacterial LPS (100 ng/ml) there was an approximately 3-fold increase in NF-κB luciferase activity compared with nontreated controls. When LPS-stimulated cells were further treated with varying concentrations of ASH–DMSO extract, a significant reduction was observed in luciferase activity at 500 and 250 μg/ml doses(**b**). Data were analyzed by 1-way analysis of variance with Bonferroni’s multiple comparison test as the post-test (***p < 0.001). Cytokines and chemokines secreted from primary microglia post-LPS challenge and the effect of ASH treatment was evaluated by commercially available array. The cytokines/chemokines whose levels were found to be significantly modulated were (**c**) FAS ligand, (**d**) interferon (IFN)-γ, (**e**) interleukin (IL)-1β (not statistically significant), (**f**) IL-4, (**g**) IL-6, (**h**) IL-17A, (**i**) monocyte chemoattractant protein-1 (MCP-1/CCL2), (**j**) macrophage inflammatory protein (MIP)-1α and (**k**) MIP-1γ, (**l**) monokine induced by IFN-γ (MIG), (**m**) the chemokine LIX, (**n**) regulated on activation, normal T cell-expressed and secreted (RANTES), (**o**) soluble TNF receptor 1 (R1), (**p**) soluble TNF receptor 2 (R2), and (**q**) chemokine (C motif) ligand 1 (XCL1). Data are represented as mean ± SEM of cytokine/chemokine levels normalized against positive controls. Values are expressed as arbitrary units (*p < 0.05; **p < 0.01; ***p < 0.001). IOD = integrated optical density; CTRL = control

To assess whether our ASH sample had the potency to inhibit the NF-κB signaling pathway, we used mouse microglial cells BV2 that were stably transfected with a NF-κB P65–luciferase reporter. BV2 cells were found to tolerate high doses of the ASH extract solubilized in DMSO, and we found no significant cell death with up to 500 μg/ml of the drug (Fig. 1a). Exposure of the cells to an inflammatory challenge (100 ng/ml *Escherichia coli* LPS) led to a 3-fold increase in luciferase activity. When the LPS-stimulated microglia were treated with varying concentrations of ASH (500, 250, 100, 10, and 1 μg/ml), there was a dose-dependent decrease in luciferase activity (*p* < 0.001) (Fig. 1b). We concluded that our ASH sample had the potency to inhibit the NF-κB signaling pathway, a potential therapeutic target in TDP-43-mediated neurodegenerative disease.

### Modulation of Cytokine/Chemokine Profile of Stimulated Primary Microglia Post-Treatment With ASH

*In vitro*, there was no significant difference between the secretory cytokine/chemokine profiles of WT or TDP-43^A315T^ microglia, with or without LPS challenge (Fig. S2a). On treating WT microglia with LPS a significant upregulation was observed in the levels of FAS ligand (2.68-fold), interferon-γ (2-fold), interleukin (IL)-1β (2.06-fold; but not statistically significant), IL-6 (13.8-fold), IL-17A (2.21-fold), monocyte chemoattractant protein-1 (2-fold), macrophage inflammatory protein (MIP)-1α (4.5-fold) and MIP-1γ (1.6-fold), monokine induced by interferon-gamma (2.8-fold), the chemokine LIX (4.68-fold), and regulated on activation, normal T cell expressed and secreted (RANTES) (2.65-fold). On the contrary, IL-4 (0.52-fold) and chemokine (C motif) ligand 1 (0.6-fold) levels were found to decrease significantly following LPS challenge. However, ASH treatment of LPS-challenged cells resulted in significant downregulation in the levels of the upregulated cytokines/chemokines and upregulation of IL-4 and chemokine (C motif) ligand 1 levels. Interestingly, even though the levels of soluble TNF receptor R1 and R2 levels were found to be elevated after LPS challenge, there was no significant alteration in the level of secreted TNF-α (data not shown) (Fig. 1c–q).

### Amelioration of Motor and Cognitive Performance in Transgenic Mice Expressing hTDP-43^A315T^

**Fig. 2:**
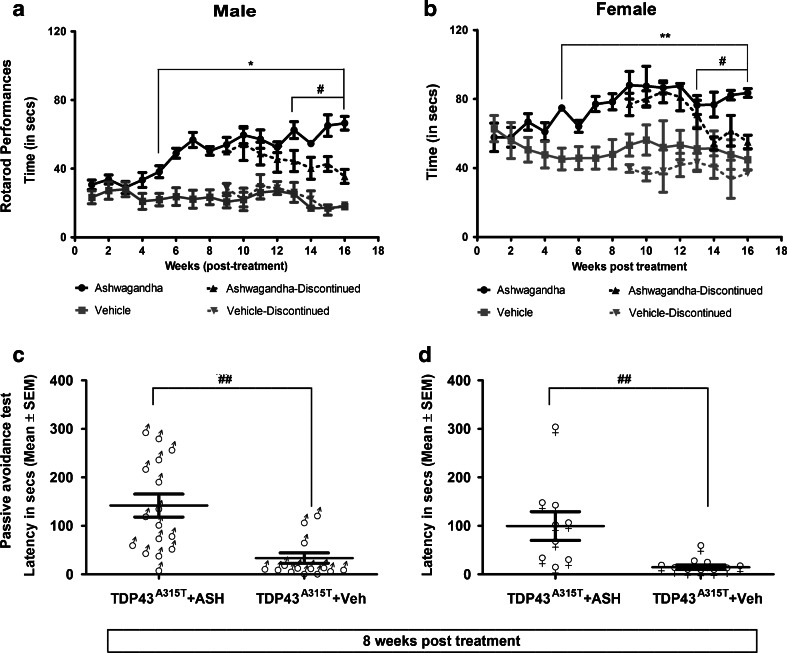
Oral Ashwagandha (ASH) administration ameliorated performance of hTDP-43^A315T^ mice on rotarod and passive avoidance tasks. ASH or vehicle (Veh)-treated male or female mice were trained to run on an accelerating rotarod (speed 3 rpm; acceleration 0.2 rpm/s) and the latency to fall was recorded. The maximum score of 3 trials for each mouse is represented. A significant increase was observed in the latency to fall in mice treated with ASH as compared with Veh-treated controls from 5 weeks post-treatment onwards. Discontinuation of ASH treatment resulted in gradual decrease in latency in both male and female mice (**a**, **b**). Data were compared using an unpaired *t* test with Welch’s correction (^#^*p* < 0.01; **p < 0.001; *p < 0.0001). After 8 weeks of treatments, male (**c**) and female (**d**) mice were subjected to the passive avoidance test to assess memory function based on the association formed with an nociceptive stimulus. The results clearly demonstrated a significant increase in memory retention in ASH-treated mice of either sex in comparison to Veh-treated controls. These data were analyzed by Kruskal–Wallis test with Dunn's multiple comparison post-test (^##^*p* < 0.01)

A suspension of ASH root extract was prepared in buffered saline at a concentration of 25 mg/ml of which 200 μl was administered orally (gavage) to hTDP-43^A315T^ every alternate day (i.e., 5 mg/per animal at one time). The treatment lasted for 8 weeks, beyond which it was continued in about 50% of the animals and discontinued in the remaining half. Control hTDP-43^A315T^ mice received equal volumes of buffered saline. When treated with ASH for a period of 8 weeks, the hTDP-43^A315T^ mice exhibited a significant increase in the latency to fall in the accelerating rotarod test when compared with Veh-treated hTDP-43^A315T^ mice (*p* < 0.0001 for males; *p* < 0.001 for females). Cessation of the treatment at 8 weeks led to a gradual decrease of performance in rotarod test for both male and female mice (*p* < 0.01 for both males and females; Fig. 2a, b). The difference observed in the latency to fall between male and female Veh-treated animals seemed to be due to the difference in their body weights. The mean body weight of male animals was always in excess of 10 to 12 g than that of the female animals, irrespective of treatment (Fig. S2b). It should be noted that ASH treatment had no effect on body weight in either male or female animals. Yet, in comparison to performance of nontransgenic animals (equal number of male and female with approximately equal body weights and equivalent age as the TDP-43^A315T^ animals) on the accelerating rotarod (Fig. S2c), only ASH-treated female mice could perform at the same level and only as long as the treatment was continued.

To further evaluate the effect of ASH on cognitive performance of hTDP-43^A315T^ mice, we used the passive avoidance test. ASH treatment of hTDP-43^A315T^ mice increased significantly the latency to enter the dark chamber, irrespective of sex (Fig. 2c, d). In contrast, the Veh-treated hTDP-43^A315T^ mice were inept at recalling the unpleasant experience encountered in the dark chamber, thereby indicating cognitive defects.

### Improvement of NMJ Innervation in hTDP-43^A315T^ Mice

**Fig. 3:**
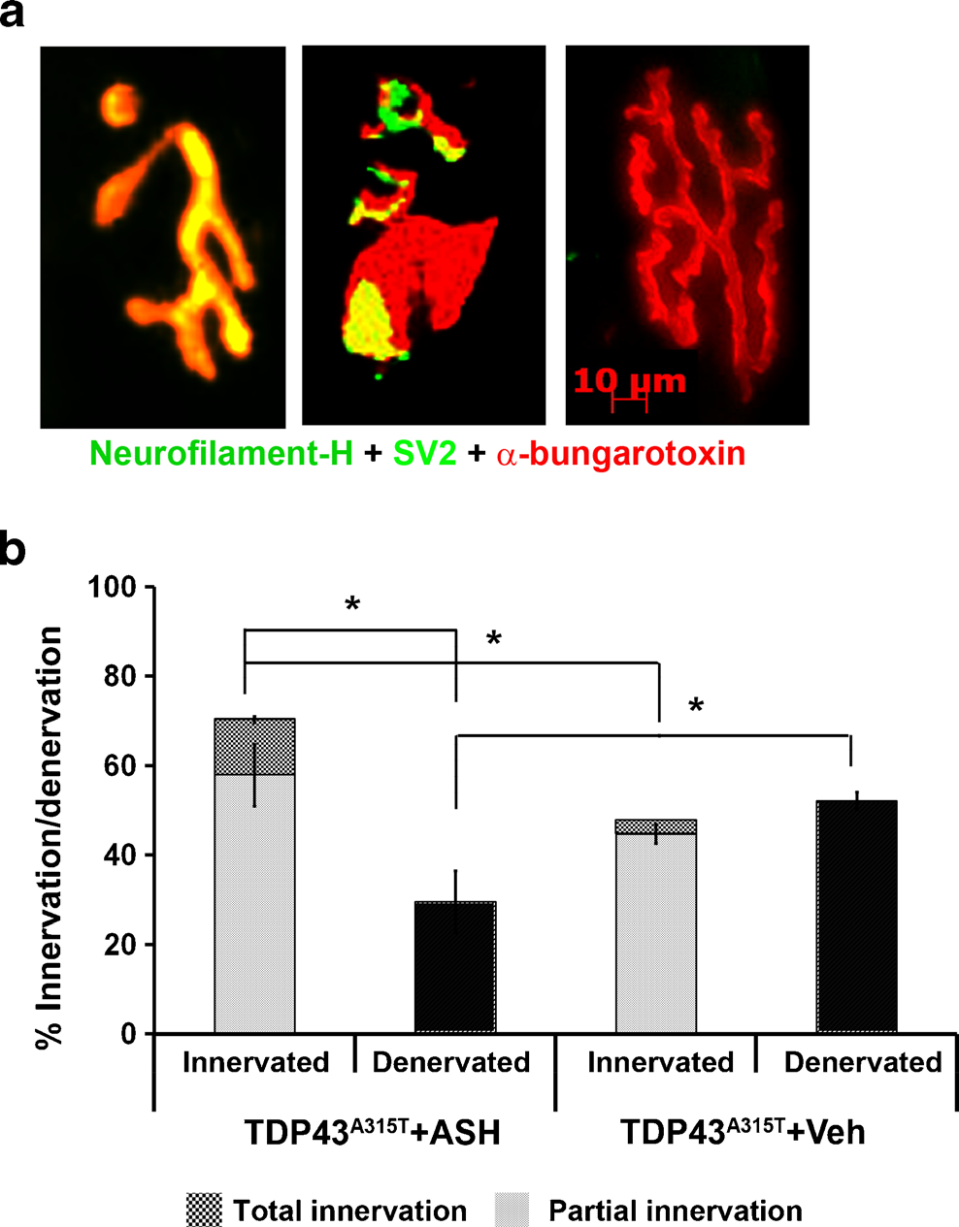
Increase of innervated neuromuscular junctions (NMJs) in Ashwagandha (ASH)-treated hTDP-43^A315T^ mice. Serial sections of gastrocnemius muscle of ASH- or vehicle (Veh)-treated hTDP-43^A315T^ mice were done with a cryostat. The sections were stained with antibodies against neurofilament-heavy chain (NF-H) and synaptic vesicle glycoprotein 2A (SV2) followed by Alexa-488 conjugated secondary antibody. Prior to mounting, the sections were briefly incubated with α-bungarotoxin conjugated with tetramethylrhodamine. The numbers of innervated, partially innervated, or denervated NMJs were counted based on the merged colored composite images of the sections (**a**). The data are represented as percent of total NMJs counted (**b**). ASH-treated mice possessed significantly more innervated NMJs than the Veh-treated controls in which roughly 50% of NMJs were denervated. Data are representative of total NMJs counted from 7 sections per mice, 3 mice per group (*n* = 1062 for ASH; *n* = 1501 for Veh) and were analyzed for statistics by 1-way analysis of variance with Bonferroni's multiple comparison post-test (*p < 0.001)

Immunohistologic analysis of gastrocnemius muscle sections to visualize NMJs revealed that in ASH-treated mice the number of innervated NMJs (complete + partial) was significantly higher than in Veh-treated animals. ASH-treated animals had significantly more number of innervated NMJs than denervated NMJs (*p* < 0.001). On comparing totally denervated NMJs, it was observed that ASH-treated mice had significantly less total denervated NMJs than Veh-treated mice (*p* < 0.001) (Fig. 3a, b). Whereas Veh-treated hTDP-43^A315T^ mice exhibited ~50% denervation of NMJs, ASH-treated mice exhibited only ~30% denervation of NMJs.

### Attenuation of Neuroinflammation in hTDP-43^A315T^ Mice

**Fig. 4:**
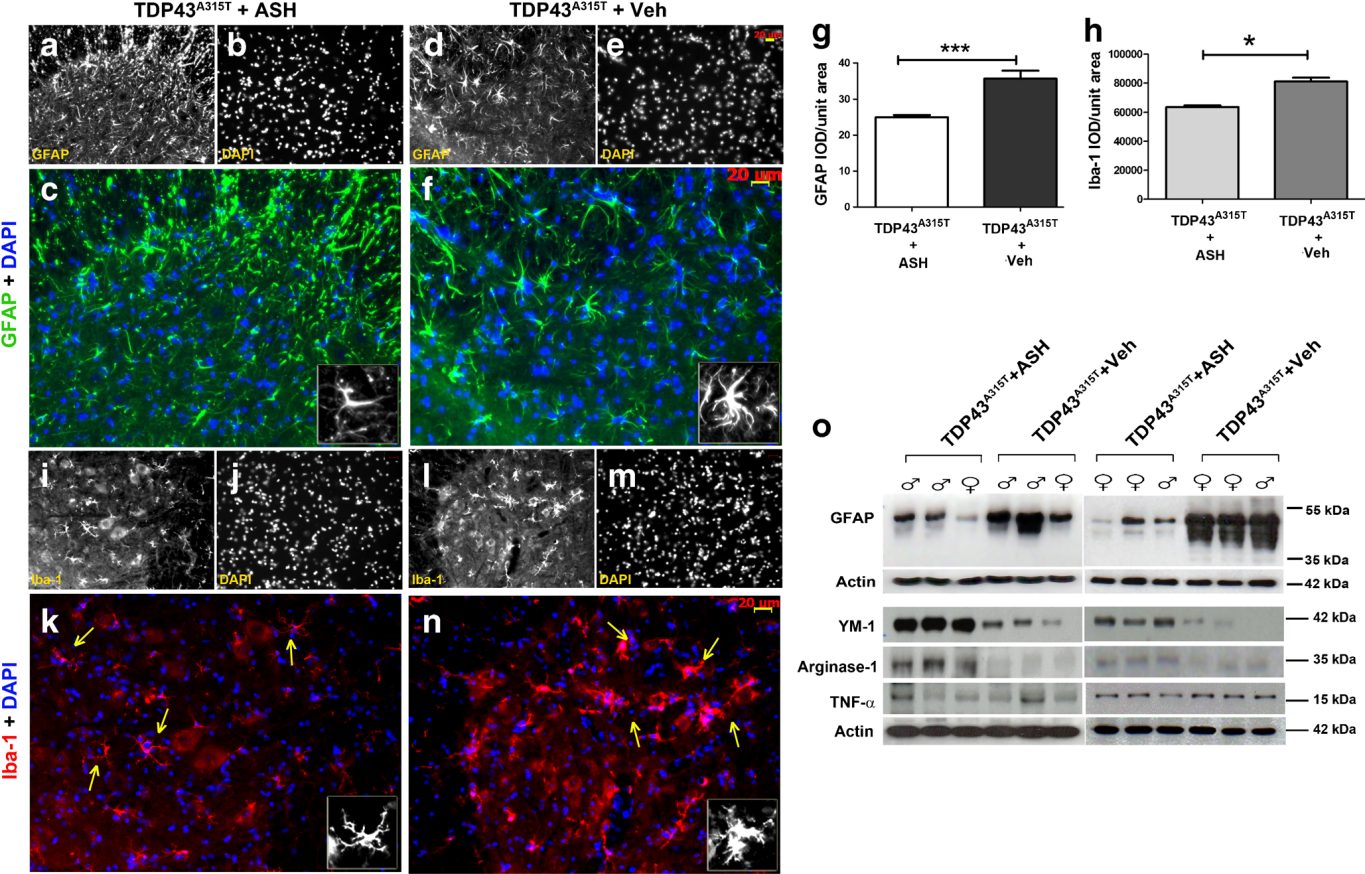
Attenuation of astrogliosis and microgliosis by Ashwagandha (ASH) treatment. Spinal cord sections from ASH- and vehicle (Veh)-treated hTDP-43^A315T^ mice were immunostained for glial fibrillary acidic protein (GFAP; astrocytic marker) or Iba-1 (microglial marker). A marked difference in the phenotype of ASH-treated (**a**–**c**) and Veh-treated (**d**–**f**) astrocytes were observed. Similarly, microglia also showed decreased activation post-ASH treatment (**i**–**k**) as compared with Veh-treated (**l**–**n**). All images are of 20× magnification. Scale bar = 20 μm. The single-channel images are in grayscale so as to give better contrast. The merged colored images are 150% enlargements. (Inset) A single cell has been enlarged to 300%. Overall integrated density (IOD) analysis of GFAP (**g**) and Iba-1(**h**) staining intensities revealed significant reduction of 28% and 23%, respectively, in the spinal cord of ASH -treated mice. Data are mean ± SEM of multiple sections of at least 3 animals per group. Statistical significance was analyzed by 2-tailed *t* test with Welch’s correction (*p < 0.05; ***p < 0.001). Western blot analysis also clearly showed more GFAP expression in the spinal cords of Veh-treated mice *vs* that in ASH-treated mice (**o**). The expression levels of 2 M2a microglial phenotypic markers (Ym-1 and arginase-1) were found to be increased in the spinal cord of ASH-treated hTDP-43^A315T^ mice when compared with Veh-treated controls. However, the tumor necrosis factor (TNF)-α levels remained the same in the 2 groups (**o**). DAPI = 4,6-diamidino-2-phenylindole

To assess the effect of ASH on astrogliosis, multiple spinal cord sections from at least 3 hTDP-43^A315T^ mice belonging to each group were immunostained for glial fibrillary acidic protein (GFAP), an intermediate filament protein present in astrocytes. The results showed distinct astrocyte morphology in the ASH- and Veh-treated spinal cord sections. Astrocytes in ASH-treated mice were thinner with long processes (Fig. 4a–c), whereas those in Veh-treated mice had thicker cell bodies but short processes (Fig. 4d–f). GFAP signal intensity analysis carried out from sections from both the groups of animals showed significantly less GFAP expression in ASH-treated mice compared with Veh-treated mice (*p* < 0.001) (Fig. 4g). Western blots also showed higher expression of GFAP in Veh-treated animals (Fig. 4o).

Morphologically, there was also a difference in microglial phenotypes of spinal cords from ASH- and Veh-treated hTDP-43^A315T^ mice. Microglia, visualized by Iba-1 staining, showed a more reactive phenotype in Veh-treated mice compared with that in ASH-treated mice. Microglia in ASH-treated mice showed small soma with distal arborization (Fig. 4i–k); in contrast, microglia in Veh-treated mice showed an increase in soma size with multiple short thick processes branching out (Fig. 4l–n). Iba-1 signal intensity analysis also corroborated a lesser intensity of staining in ASH-treated animals (*p* < 0.05) (Fig. 4h). Ym-1 and arginase-1 are well-reported phenotypic markers for M2a microglia. In our samples, we saw an increased expression of both of these proteins in ASH-treated animals compared with Veh-treated animals. However, we failed to detect any difference in the levels of TNF-α, a secreted cytokine from type M1 and M2b microglia (Fig. 4o).

### Reduced p65 NF-κB Levels in the Spinal Cord of hTDP-43^A315T^ Mice

**Fig. 5:**
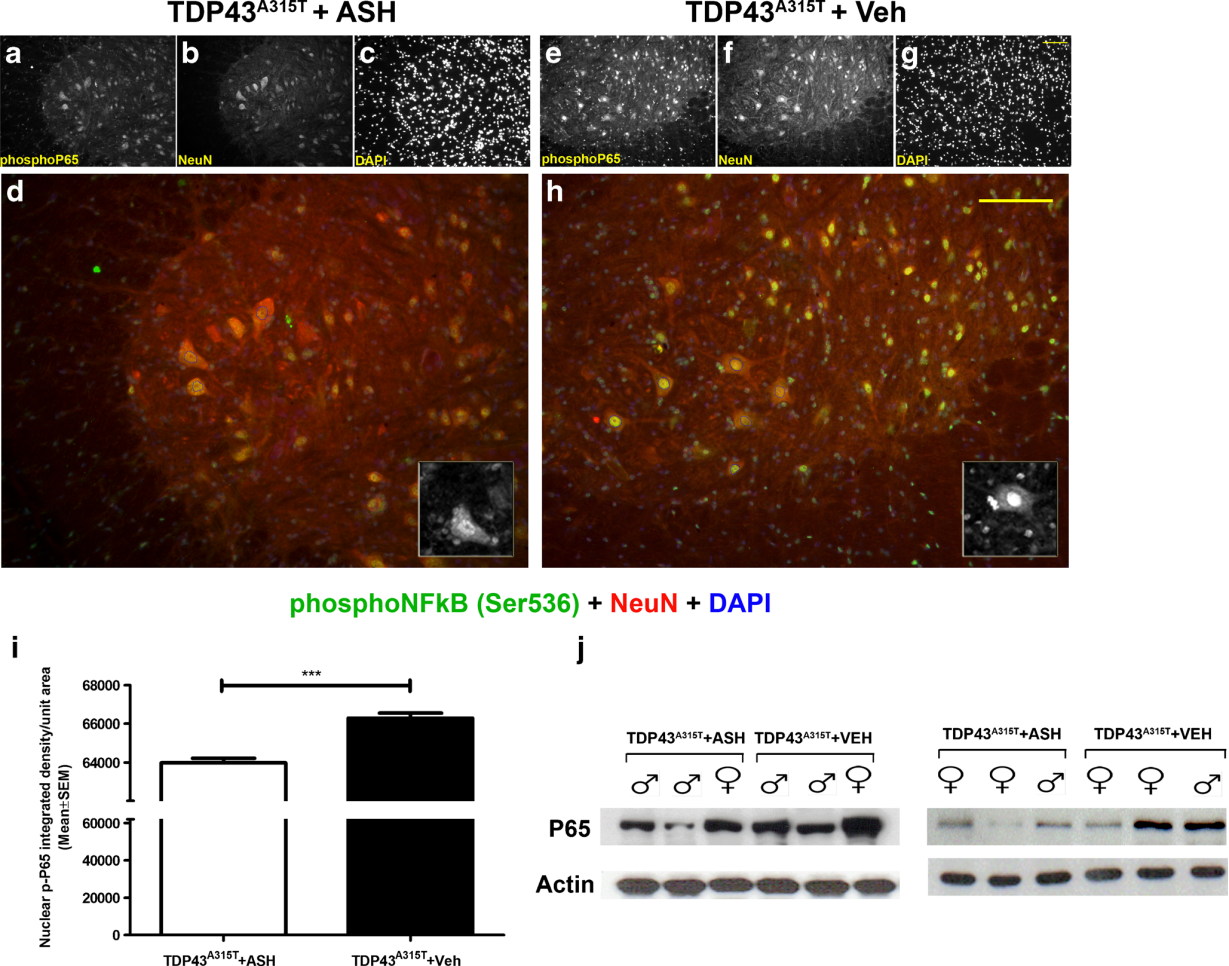
Reduction of p65 nuclear factor kappa B (NF-κB) activation in spinal cord of Ashwagandha (ASH)-treated hTDP-43^A315T^ mice. Spinal cord sections from ASH- and vehicle (Veh)-treated hTDP-43^A315T^ mice were immunostained for detection of phophorylated p65 NF-κB. A significantly weaker staining signal was observed in the nucleus of neurons (including motor neurons) from the ASH-treated hTDP-43^A315T^ mice (**a**–**d**) compared with Veh-treated hTDP-43^A315T^ mice (**e**–**h**). All images are of 20× magnification. Scale bar = 20 μm. The single-channel images are grayscale so as to give better contrast. The merged colored images are 150% enlargements. (Inset) A single cell with phosphoP65 staining has been enlarged to 300%. To further confirm this, the nuclear signal intensities from neurons of both groups were measured using ImageJ after demarcation of the nuclear area using 4,6-diamidino-2-phenylindole (DAPI) in Adobe Photoshop (outline drawn in blue). Upon comparison there was significantly lower intensity of nuclear signal in ASH-treated mice than in Veh-treated mice (**i**). Data are mean ± SEM of signal intensities per unit nuclear area from at least 6 sections per mouse, 3 mice per group. Data were analyzed by 2-tailed *t* test with Welch’s correction (***p < 0.001). Immunoblot performed from whole spinal cord lysate also revealed lower expression levels of P65 NF-κB protein in ASH-treated mice than in Veh-treated controls (**j**)

Spinal cord sections from both ASH- and Veh-treated mice were immunostained for detection of phophorylated p65 NF-κB. On comparing images, captured at same exposure levels, results showed that the nucleus of neurons in ASH-treated mice (Fig. 5a–d) yielded a lesser signal than that observed in Veh-treated animals (Fig 5e–h). Integrated density of the signal, analyzed from individual neurons after background removal using Image J, showed a significant increase in presence of phopho p65 NF-κB in neuronal nuclei of Veh-treated mice (*p* < 0.001) (Fig. 5i). Immunoblot analysis from spinal cord extract also showed an increase in total p65 NF-κB protein in Veh-treated mice (Fig. 5j).

### Rescue of TDP-43 Mislocalization and Aggregation

**Fig. 6:**
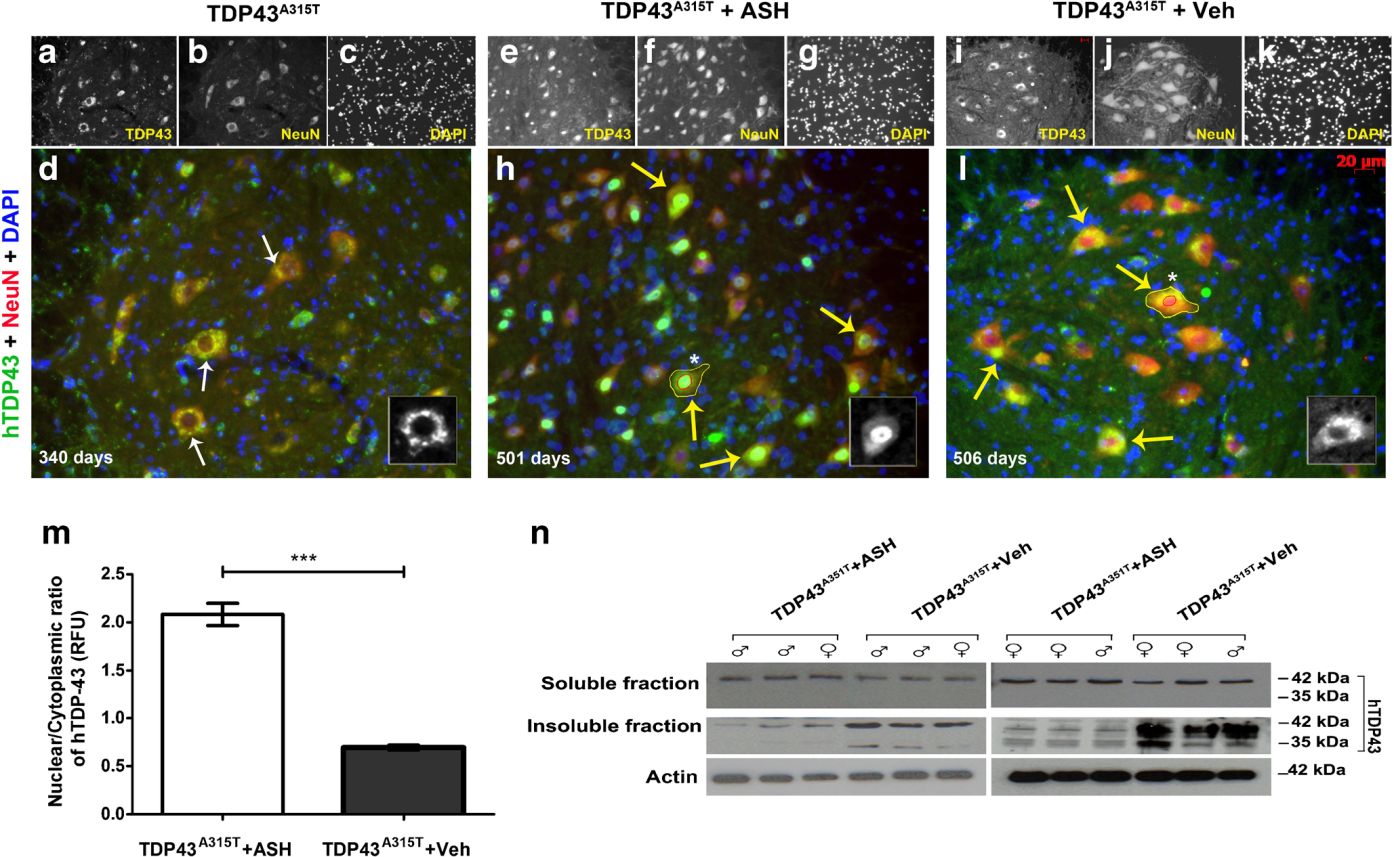
Alleviation of human transactive response DNA binding protein 43 (hTDP-43) mislocalization and aggregation in Ashwagandha (ASH)-treated hTDP-43^A315T^ mice. In hTDP-43^A315T^ mice, there is a clear nuclear depletion of hTDP-43 occurring in spinal motor neurons at about 11 months of age (**a**–**d**). Therefore, ASH treatment was initiated after this age. After 8 weeks of treatment, the spinal cord of hTDP-43^A315T^ mice (~500 days old) exhibited intense immunodetection of hTDP-43 in the nucleus of motor neurons (**e**–**h**). In contrast, the Veh-treated mice exhibited diffused hTDP-43 in the cytoplasm of spinal cord neurons (**i**–**l**). All images are of 20× magnification. Scale bar = 20 μm. The single-channel images are grayscale so as to give better contrast. The merged colored images are 150% enlargements. (Inset) A single cell with hTDP-43 staining has been enlarged to 300%. Nuclear:cytoplasmic hTDP-43 stain intensity was calculated from motor neurons (neurons with diameter ≥4 μm) present in ventral horn of spinal cord sections using ImageJ after demarcation of the nuclear (outline drawn in red) and whole cell area (outline drawn in yellow) using 4,6-diamidino-2-phenylindole (DAPI) in Adobe Photoshop. Results showed a significant increase of about 3.5-fold in the nuclear to cytoplasmic ratio in ASH-treated spinal motor neurons compared with Veh-treated samples (**m**). Data were analyzed by 2-tailed *t* test with Welch’s correction (***p < 0.001). On subjecting detergent-soluble and insoluble fractions of spinal cord lysate to immunoblotting, no major difference was observed in the soluble hTDP-43 levels between the 2 groups. However, hTDP-43 was present at lower levels in the insoluble fraction of ASH-treated hTDP-43^A315T^ mice when compared with Veh-treated hTDP-43^A315T^ mice. ASH treatment caused also a reduction in levels of the toxic 35-kDa TDP-43 species (cleaved fragment/splice variant) (**n**)

Transgenic mice expressing hTDP-43^A315T^ start exhibiting mislocalization of TDP-43 into the cytoplasm of spinal motor neurons at approximately 8 months of age. At 12 months of age, almost all spinal neurons showed a lack of nuclear staining for hTDP-43 (Fig. 6a–d). On completion of the treatment schedule after 8 weeks, the average age of the male mice was of ~505 days and that of the female mice of ~398 days. Post-ASH treatment, hTDP-43 protein was mostly detected in the nucleus of spinal neurons (Fig. 6e–h), whereas hTDP-43 had a diffused cytoplasmic localization in spinal neurons of Veh-treated animals (Fig. 6i–l). A nuclear/cytoplasmic ratio of immunofluorescent signal from 21 lumber spinal cord sections of 3 mice per group, analyzed using ImageJ software, showed a significantly increased nuclear immunostaining of hTDP-43 (*p* < 0.001) (Fig. 6m). This redistribution was corroborated by immunoblot data showing the presence of increased full-length hTDP-43 (and the toxic 35-kDa cleaved fragment/splice variant) in the detergent-insoluble fractions of spinal cord of Veh-treated mice compared with the ASH-treated mice (Fig. 6n). In the brain, cortical neurons displayed similar results. In ASH-treated hTDP-43^A315T^ mice, hTDP-43 had predominant nuclear localization in cortical neurons. Conversely, in Veh-treated mice, hTDP-43 was predominantly cytosolic (Fig. S2d, e).

### Reduction of Peripherin Expression

The intermediate filament protein peripherin has been shown to be involved in neurite elongation at developmental stage and axonal regeneration but could also be responsible for protein aggregation and motor neuron death in ALS [42]. In Veh-treated transgenic mice, peripherin was found to be prominently expressed. However, in the spinal cord of hTDP-43^A315T^ mice treated with ASH, there was a marked reduction in peripherin levels (Fig. S2f). Note that peripherin levels in female hTDP-43^A315T^ mice were higher than in male mice, irrespective of the treatment.

### Nuclear TDP-43 Redistribution in Cell Culture System is Mediated by Secreted Factors From ASH-Treated Microglial Cells

**Fig. 7:**
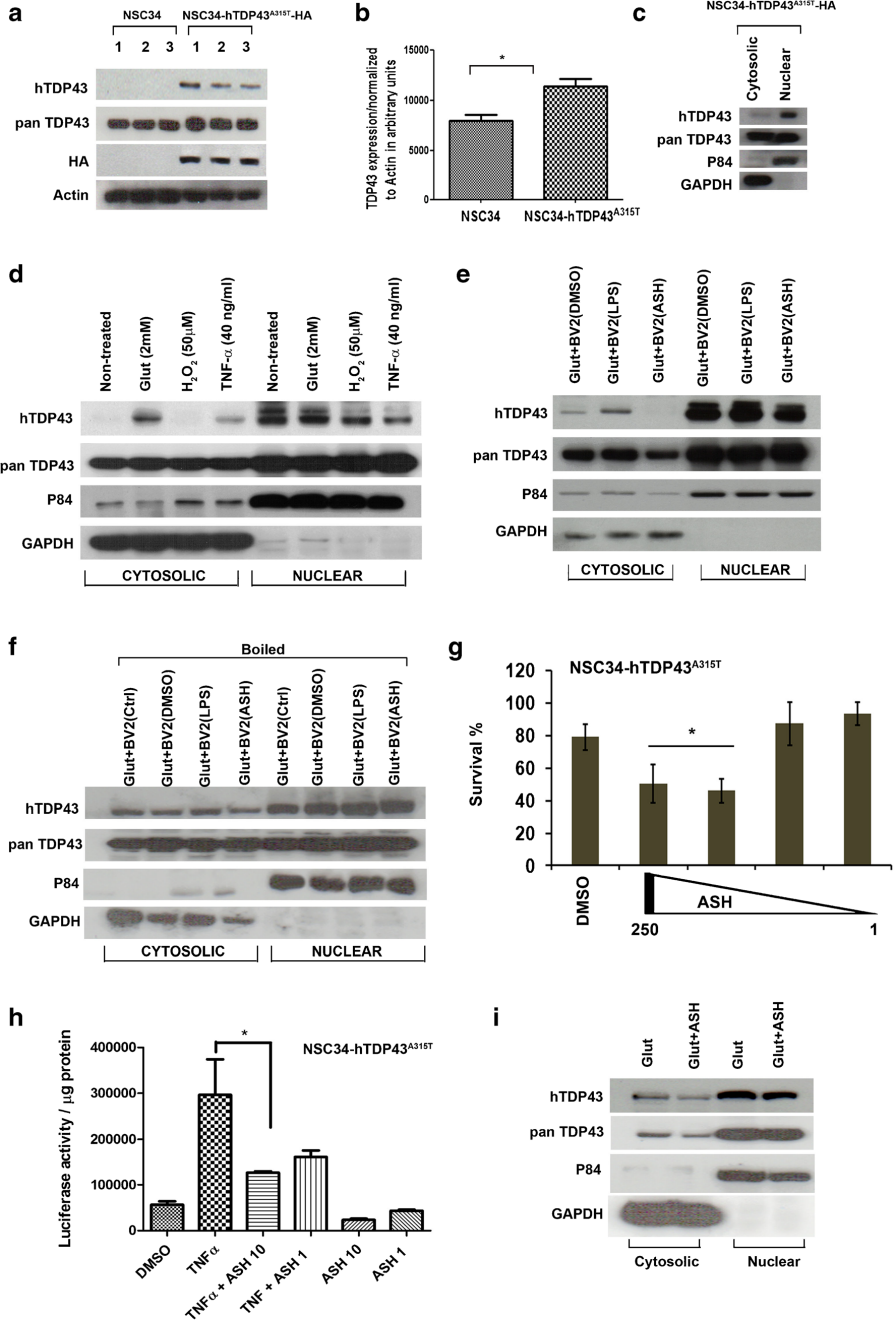
Evidence that nuclear redistribution of human transactive response DNA binding protein 43 (hTDP-43) may result from soluble factors released from microglia in response to Ashwagandha (ASH) treatment. To study the role of microglial factors on TDP-43 redistribution in motor neurons *in vitro*, a model was devised wherein motor neuron-like immortalized cell line NSC34 was stably transfected with hTDP-43^A315T-HA^ plasmid DNA (**a**). The stable cells showed about 30% increase in TDP-43 expression (**b**). Data were analyzed by *t* test with Welch’s correction (*p < 0.05). hTDP-43 was found to be localized predominantly inside the nucleus of these stably transfected cells (**c**). Various treatments were tested for their potency to induce hTDP-43 cytoplasmic mislocalization. The cells were treated with either glutamate (2 mM) or H_2_O_2_ (50 μM) or tumor necrosis factor (TNF)-α (40 ng/ml). Glutamate was the most effective inducer of hTDP-43 mislocalization (**d**). Mouse microglial cell line BV2 was treated with dimethyl sulfoxide (DMSO), lipopolysaccharide (LPS; 100 ng/ml), or ASH (250 μg/ml) in solution in DMSO. The conditioned media from these cells were used to treat glutamate-pretreated stably transfected NSC34 cells. The conditioned media from BV2 cells induced cytoplasmic mislocalization of hTDP-43 in NSC34 cells. However, hTDP-43 was found to be cleared off from the cytosolic fraction of NSC34 cells exposed to conditioned media from ASH-treated microglia (**e**). Heat inactivation of the conditioned media prior to exposure to NSC34 cells abolished the cytoplasmic clearance of hTDP-43 by media of ASH-treated BV2 cells (**f**). The stably transfected NSC34 cells were found to tolerate exposure to ASH solution poorly. On incubation with 250 and 100 μg/ml ASH, the viability of these NSC34 cells was significantly reduced to 50% within 6 h. However, dosages of 10 and 1 μg/ml were found to be tolerated (**g**). Data were analyzed by 1-way analysis of variance (ANOVA) with Bonferroni’s post-test (**p < 0.01). The efficacy of 10 and 1 μg/ml doses of ASH on reduction of P65 activity in stably transfected NSC34 cells were studied by luciferase assay. Results showed that the 10 μg/ml was sufficient to significantly reduce P65 activity post-TNF-α challenge (**h**). Data were analyzed by 1-way ANOVA with Bonferroni’s post-test (***p < 0.05). However, ASH treatment of NSC34 cells at 10 μg/ml failed to eliminate the cytosolic mislocalization of hTDP-43 induced by glutamate (**i**). HA = Hemagglutinin antigen ; GAPDH = glyceraldehyde 3-phosphate dehydrogenase

To further address whether the nuclear redistribution of TDP-43 was due to a direct effect of ASH on neurons or indirect effect via microglial cells, we used motor neuron-like cells NSC34 that were stably transfected to express human TDP-43^A315T^. TDP-43 was overexpressed in these cells by about 30% in comparison with nontransfected cells, as shown by immunoblotting with pan TDP-43 antibody (*p* < 0.05) (Fig. 7a, b). However, it was observed that the hTDP-43^A315T^ remained compartmentalized in the nuclear fraction in unstimulated transgenic cells (Fig. 7c). To induce hTDP-43 mis-localization into the cytoplasm the transfected cells were challenged with excitotoxic, oxidative, or inflammatory stimuli with 2 mM glutamate, 50 μM H_2_O_2_, and 40 ng/ml TNF-α, respectively. Immunoblot analysis showed that TDP-43 was detected in cytosolic fractions from both glutamate- and TNF-α-treated transfected cells but not in H_2_O_2_-treated cells (Fig. 7d). As the cytoplasmic levels of TDP-43 were higher with glutamate treatment, further studies were conducted using this paradigm. On culturing glutamate pretreated NSC34–hTD-P43^A315T^ cells in presence of conditioned media from mock-treated or LPS-stimulated microglial cells, it was observed that hTDP-43 could be detected in cytoplasmic fraction. However, in cells cultured with conditioned media from ASH-treated microglia, there was almost no detectable hTDP-43 in the cytoplasmic fraction (Fig. 7e). Note that to generate conditioned media, microglial cells were treated with 250 μg/ml ASH dissolved in DMSO. To confirm the role of soluble factors released from microglial cells on this TDP-43 redistribution phenomenon, the same experiment was repeated with conditioned media which was first heated to 70 °C to denature factors. Subsequent immunoblot analysis of cytosolic and nuclear fractions showed uniform presence of hTDP-43 in cytosol of cells from all three groups, thereby suggesting a disruption of the redistribution (Fig. 7f).

We further investigated whether there was a direct effect of ASH on NSC34. On direct treatment of NSC34–hTDP-43^A315T^ with 250 μg/ml ASH solution, almost 50% cell death was observed just after 4 h of incubation. By reducing the dose to 100 μg/ml no significant improvements were observed, but on further lowering the concentrations (10 and 1 μg/ml) the cells seemed to tolerate ASH (Fig. 7g). To test whether these low ASH concentrations could affect P65 NF-κB activity in NSC34–hTDP-43^A315T^ cells, the P65 luc reporter was transfected, as described in the “Materials and Methods” section. The NSC34–hTDP-43^A315T^-P65 luc cells were then treated with low dosage of ASH, with or without stimulation with recombinant TNF-α. Luciferase assay from these cells clearly showed that these doses were sufficient to reduce significantly P65 activity post-TNF-α treatment (*p* < 0.01) (Fig. 7h). Interestingly, it was also observed that direct ASH treatment of glutamate-pretreated NSC34–TDP-43^A315T^ cells did not alter TDP-43 distribution (Fig. 7i).

## Discussion

There is compelling evidence that the NF-κB signaling pathway may represent a rational therapeutic target for ALS. For instance, a number of ALS-linked genes encode proteins that may interact with the NF-κB signaling pathway: 1) TDP-43 and FUS can bind and activate p65 NF-κB [12, 30]; 2) ALS-linked mutations have been discovered in the optineurin gene, which encodes a protein activating the suppressor of NF-κB [31]; 3) mutations in valosin-containing protein activates NF-κB signalling [32, 33]; 4) overt inflammation was present in mice deficient for progranulin, a negative regulator of NF-κB activity [34]; 5) p62 (*SQSTM1*) can be associated with exacerbated inflammatory responses [35] and mutations in ALS/FTLD increase p62 levels [36]; 6) NF-kB signaling pathway is upregulated in ALS induced pluripotent stem cell-derived motor neurons [37]; 7) ablation of NF-κB signaling in microglia extended survival of superoxide dismutase 1 mutant mice [38].

To attenuate activation of the NF-κB signaling pathway in a transgenic mouse model of ALS, we tested an extract of *W. somnifera* (ASH) that had the potency to reduce in a dose-dependent fashion the activity of NF-κB P65–luciferase reporter in the microglial cultured BV2 cells (Fig. 1). The anti-inflammatory properties of ASH were further demonstrated by it role in inhibiting proinflammatory cytokines/chemokines released by primary mouse microglia as a result of an inflammatory stimulus. Among the variety of cytokines and chemokines affected, of particular interest are RANTES, IL-6, IL-17, and MIP-1α, whose circulating levels in serum and/or cerebrospinal fluid are reported to be elevated in ALS [39–42]. Likewise, soluble TNF receptors R1 and R2 levels are also reported to be elevated in plasma of patients with ALS [43]. IL-4 levels have been reported to be negatively correlated with IL-6 levels in cerebrospinal fluid of ALS cases. Thus, if IL-6 level increases, IL-4 decreases [44]. *In vitro*, ASH restored LPS-induced downregulation of microglial IL-4 secretion and thus may contribute to modulation of inflammatory processes in multiple ways.

Transgenic mice expressing hTDP-43^A315T^ genomic fragment used in this study exhibited age-associated pathologic changes, including TDP-43 proteinopathy, cognitive deficits, and motor dysfunction [24]. When administered orally by gavage in hTDP-43^A315T^ transgenic mice >1 year of age, ASH was found to ameliorate motor performance on rotarod test, as well as cognitive function, as determined by passive avoidance test. Moreover, ASH ameliorated muscle innervation, as well as pathological changes. Peripherin is an intermediate filament protein whose accumulation is a pathological hallmark of ALS and transgenic mouse studies demonstrated that sustained peripherin overexpression can cause motor neuron death [45, 46]. Peripherin levels are upregulated in the hTDP-43^A315T^ transgenic mice [24], and ASH treatment caused a marked reduction in peripherin levels.

Of particular interest was the finding that ASH led to redistribution of cytoplasmic hTDP-43 to the nucleus in spinal motor neurons and in brain cortical neurons of TDP-43^A315T^ transgenic mice after 8 weeks of treatment (Fig. 6a–d,f,g ). Moreover, ASH treatment reduced the levels of hTDP-43 recovered in the detergent-insoluble fraction of spinal cord (Fig. 6e). Thus, ASH treatment may confer protection by rescuing both the mislocalization and aggregation of hTDP-43. It is noteworthy that ASH treatment led to a significant reduction in levels of phospho-NF-κB in the nucleus of spinal motor neurons of hTDP-43^A315T^ mice (Fig. 5a–c). This observation provides further evidence of a link between inflammation and TDP-43 pathology. This is in line with our recent report that chronic induction of inflammation by LPS treatment exacerbated TDP-43 proteinopathy in hTDP-43^A315T^ mice [47].

The neuroprotective effects of ASH in this mouse model led us to further investigate whether ASH exerted benefits directly on neurons or indirectly on neurons via the action of the compound on other CNS cell types like glial cells. To address whether ASH may exert a direct protective effect on neurons, we used NSC34 motor neuron-like cells that were stably transfected to express hTDP-43A^315T^. Glutamate induced excitotoxic stress and cytoplasmic mislocalization of hTDP-43 in NSC34 cells. However, ASH treatment at10 μg/ml did not rescue this phenomenon (Fig. 7i), but treatment of glutamate-pretreated NSC34–hTDP-43^A315T^ cells with conditioned media from ASH-treated microglial cells corrected the cytoplasmic mislocalization of hTDP-43. The factors from ASH-treated microglia involved in hTDP-43 redistribution into nucleus were inactivated by heat (Fig. 7f). Therefore, according to these *in vitro* results, the beneficial effects of ASH on neuronal pathology likely arise indirectly from the action of the drug on glial cells. Actually, it is well established that glial cells contribute to the pathogenesis of ALS [48]. Astrocytes have been reported in multiple studies to be implicated in inducing motor neuron death [49, 50]. Yet, astrocyte activation in response to factors released from damaged motor neurons may also trigger the release of nerve growth factor and other antioxidative enzymes that confer neuroprotection [51]. The role of microglia is also complex. In response to neurodegeneration or to accumulations of misfolded proteins, microglia can proliferate and adopt an activated state with secretion of several factors that may be either beneficial or detrimental to neurons [52, 53]. Hence, microglial responses can vary from neuroprotective (M2a state) to injurious/toxic states (M1, classically activated) [54]. In our study, after 8 weeks of ASH treatment, we observed a significant reduction in GFAP expression in the spinal cord of hTDP-43^A315T^ mice, as well as reduction of Iba-1 immunoreactivity in microglia, although many of these cells did not assume a quiescent morphology after ASH treatment. Immunoblotting of spinal cord extracts revealed an upregulation in levels of YM-1 and arginase-1, which are markers of microglial M2a phenotype.

In conclusion, our results demonstrated beneficial effects of ASH treatment on behavioral and pathologic phenotypes of transgenic mice expressing hTDP-43^A315T^. It remains to be determined what components in ASH are responsible for the therapeutic effects. One active molecule in ASH is Withaferin A, a steroid lactone known to act as NF-κB inhibitor and which has been shown to confer protection in 2 different mouse models of ALS [12, 26]. Nonetheless, other components present in ASH may also confer protection. For instance, sominone, a metabolite of withanoside IV has been shown to promote neurite outgrowth and to improve learning and memory deficits in mice [55]. An extract of *W. somnifera* was also effective in reversing behavioral deficits and amyloid plaque burden in a mouse model of Alzheimer’s disease [56]. In addition to diseases of the ALS/FTLD spectrum, TDP-43 pathology has been reported in other neurologic disorders including Alzheimer’s disease, Parkinson’s disease, Lewy body disease, cerebral ischemia, and hippocampal sclerosis [57]. ASH is a natural health product, easily accessible, and has been well tolerated in previous studies. Nonetheless, there are some drawbacks to such a therapeutic approach. As with other plant extracts, it is still not clear which components of ASH are responsible for what specific beneficial effects and whether the benefits are due to compounds acting alone or synergistically with others in whole extracts. Moreover, huge variations in composition of ASH extracts may occur as a result of variations in the source of *W. somnifera* or methods of root extract preparations. Based on the findings presented here, it is of interest to further investigate whether ASH extract or it constituents might be effective in conferring therapeutic effects in TDP-43 proteinopathies like ALS and FTLD.

## Electronic supplementary material

Below is the link to the electronic supplementary material.

ESM 1 (PDF 872 kb)

ESM 2 (PDF 484 kb)
