# Supplementary material for: Withania somnifera Reverses Transactive Response DNA Binding Protein 43 Proteinopathy in a Mouse Model of Amyotrophic Lateral Sclerosis/Frontotemporal Lobar Degeneration
Source: Neurotherapeutics. 2016 Dec 7;14(2):447–62. doi: 10.1007/s13311-016-0499-2 (PMC5398980; doi:10.1007/s13311-016-0499-2)
Supplement: Supplementary file 1 — (PDF 872 kb) [file 13311_2016_499_MOESM1_ESM.pdf]

Supplementary Figures for the paper entitled "*Withania somnifera* reverses TDP-43 proteinopathy in a mouse model of ALS/FTLD"

Kallol Dutta, Priyanka Patel, Reza Rahimian, Daniel Phaneuf and Jean-Pierre Julien

Legends

Fig S1: Figure shows the date of birth of all the animals that were used for the study. Animals were randomly assigned numbers from the animal facility so as to mask identity of treatment group from experimenter. Animal #61-71 and 73-77 were male $TDP43^{A315T}$ mice which received Ashwagandha; # 87-91 and 93-101 were male $TDP43^{A315T}$ mice which received Vehicle; # 323, 324, 357, 366, 369, 384, 385, 392, 393 were female $TDP43^{A315T}$ mice which received Ashwagandha; and # 394, 397, 398, 400, 410, 411, 416, 417, 419, 425 were female $TDP43^{A315T}$ mice which received Vehicle.

Figure S2: Cytokine array performed from culture supernatant of wild-type (WT) and $TDP43^{A315T}$ primary microglia treated either with DMSO or LPS (100 ng/mL) did not show any variation where each dot represents one cytokine. The 4 dots on upper left corner are the 4 positive controls with which data are normalized. As can be clearly visualized, corresponding dots in either DMSO-treated or LPS-treated groups did not show any inter-group variation, thereby indicating WT as well as $TDP43^{A315T}$ behave similarly *in vitro* (a). Body weight records of ASH and Veh-treated $TDP43^{A315T}$ male and female mice shows males were considerably heavier. However, ASH treatment did not elicit any change in body weight (b). Basal rotarod performance record of non-transgenic (wild-type) mice (3 male; 3 female) recorded over a period of 6 months. Age of the animals is similar to that of those utilized for our study (c). The beneficial effect of ASH

on nuclear redistribution of hTDP-43 was also observed in brain cortical neurons (d-e). Magnification = 63X; scale = 10μ. Immunoblotting also revealed a marked reduction of peripherin levels in spinal cord lysates of ASH-treated transgenic mice when compared to age and sex-matched controls. However, females had higher levels of peripherin, irrespective of treatment, than males (f).

Fig S1

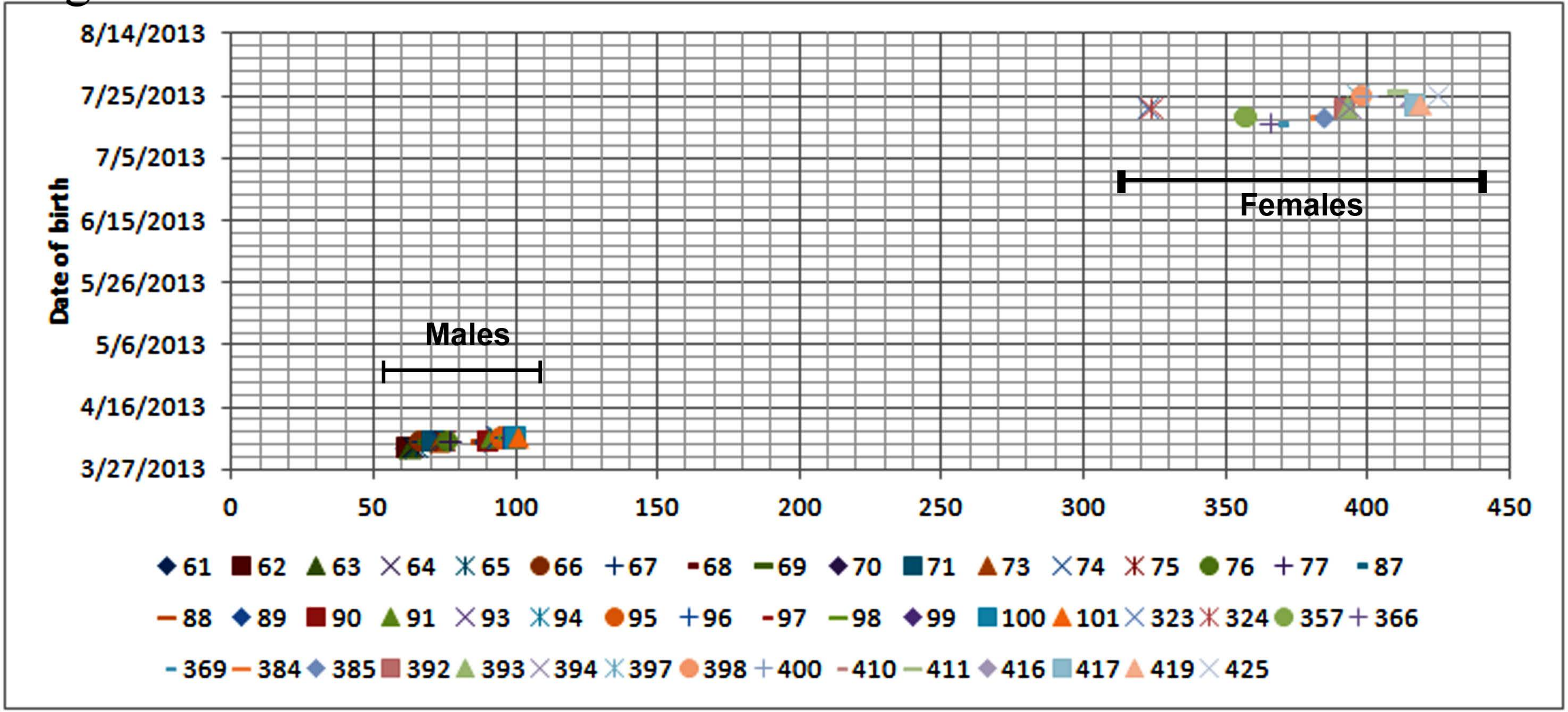

Date of birth
8/14/2013
7/25/2013
7/5/2013
6/15/2013
5/26/2013
5/6/2013
4/16/2013
3/27/2013
0
50
100
150
200
250
300
350
400
450
Females
Males
61 62 63 64 65 66 67 68 69 70 71 73 74 75 76 77 87
88 89 90 91 93 94 95 96 97 98 99 100 101 323 324 357 366
369 384 385 392 393 394 397 398 400 410 411 416 417 419 425

Fig S2

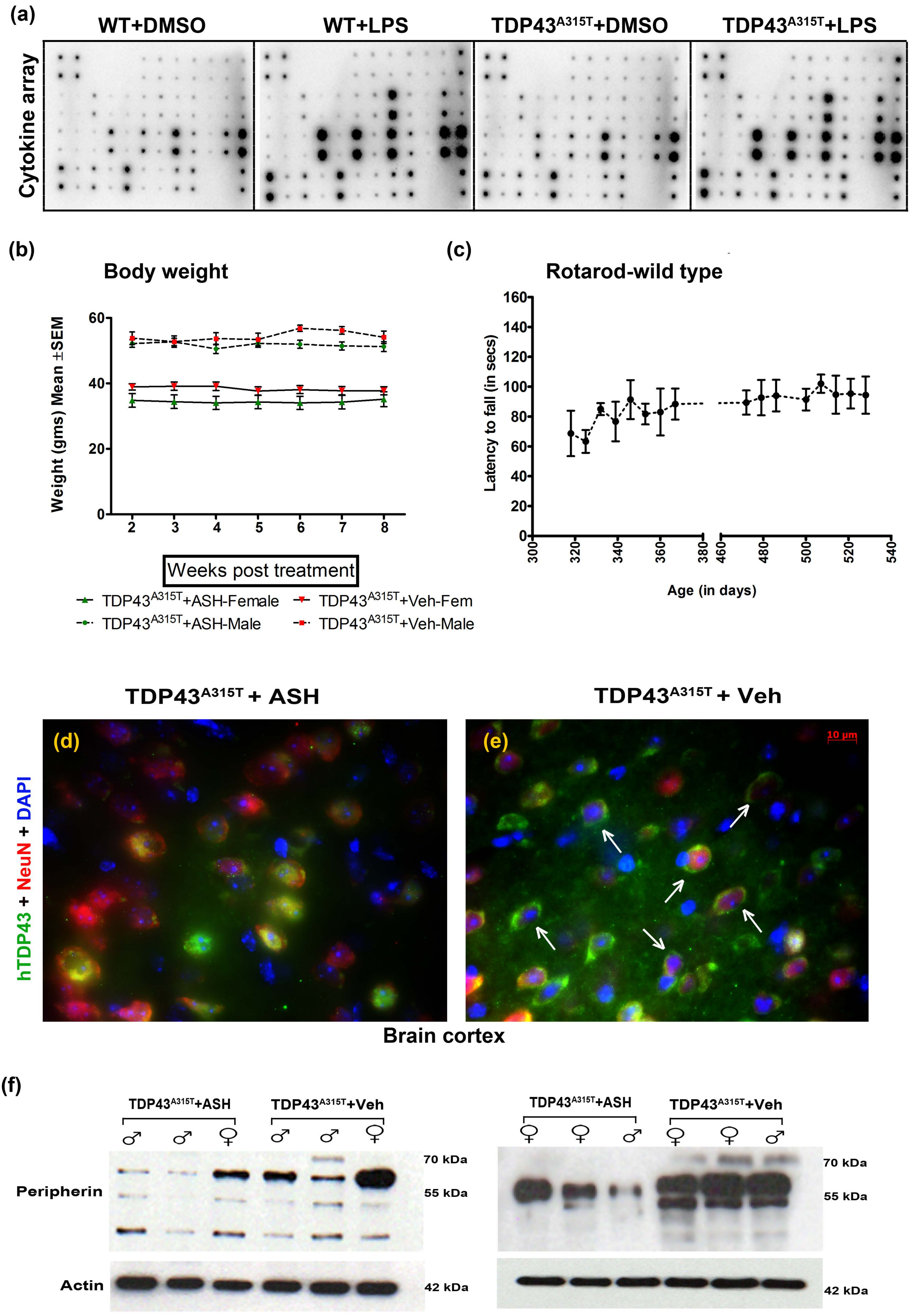
(a)
WT+DMSO
WT+LPS
TDP43A315T+DMSO
TDP43A315T+LPS
Cytokine array
(b)
Body weight
Weight (gms) Mean ±SEM
Weeks post treatment
TDP43A315T+ASH-Female
TDP43A315T+ASH-Male
TDP43A315T+Veh-Fem
TDP43A315T+Veh-Male
(c)
Rotarod-wild type
Latency to fall (in secs)
Age (in days)
TDP43A315T + ASH
TDP43A315T + Veh
(d)
(e)
10 µm
hTDP43 + NeuN + DAPI
Brain cortex
(f)
TDP43A315T+ASH
TDP43A315T+Veh
Peripherin
Actin
70 kDa
55 kDa
42 kDa
