# Supplementary material for: Withania somnifera Reverses Transactive Response DNA Binding Protein 43 Proteinopathy in a Mouse Model of Amyotrophic Lateral Sclerosis/Frontotemporal Lobar Degeneration
Source: Neurotherapeutics. 2016 Dec 7;14(2):447–62. doi: 10.1007/s13311-016-0499-2 (PMC5398980; doi:10.1007/s13311-016-0499-2)
Supplement: Supplementary file 2 — (PDF 484 kb) [file 13311_2016_499_MOESM2_ESM.pdf]

Avertissement : ce formulaire n'est pas pris en charge par la version actuelle d'Acrobat ou d'Adobe Reader.

Mise Ã niveau vers la derniÃ¨re version pour une prise en charge complÃ¨te.
